# Molecular Biology and Epidemiology of Neurotropic Viruses

**DOI:** 10.7759/cureus.9674

**Published:** 2020-08-11

**Authors:** Abba Musa Abdullahi, Shah T Sarmast, Romil Singh

**Affiliations:** 1 Neurology/Neuroscience, Hasiya Bayero Paediatric Hospital, Kano, NGA; 2 Neurology, California Institute of Behavioral Neurosciences & Psychology, Fairfield, USA; 3 Internal Medicine, Metropolitan Hospital, Jaipur, IND

**Keywords:** neurotropic viruses, neuroinvasive, neurovirulence, genome, replication, prevalence and incidence

## Abstract

Neurotropic viruses are those viruses that can cause central nervous system (CNS) diseases with both neuroinvasive and neurovirulence properties. It comprises a wide range of viruses, including herpes simplex virus, poliovirus, enteroviruses, parechovirus, West Nile virus, Japanese encephalitis virus, measles, and mumps viruses among others. Some of these viruses are highly neuroinvasive and neurovirulent, while others are weakly neuroinvasive and neurovirulent. Moreover, some of them, like herpes simplex viruses, are highly neuroinvasive but weakly neurovirulent for the peripheral nervous system and highly neurovirulent but weakly neuroinvasive for the central nervous system. All these disparities are a result of differences in their genomic constitution, associated vectors, geographical region, and environmental factors. Therefore, a successful intervention will be almost impossible without a clear understanding of the molecular biology and epidemiology of these viruses. Thus, we conducted a review of the published studies on the molecular biology and epidemiology of the common neurotropic viruses to make the viral genetic makeup more understandable for targeted intervention and provide the morbidity and mortality data of the different neurotropic viruses for more serious action.

## Introduction and background

A large number of viruses have been implicated in many neurological diseases in different parts of the world which were shown to disrupt the anatomy and physiology of the central nervous system (CNS), resulting in deformity and a high fatality rate. Therefore, effective therapeutic measures are needed to address the problems caused by these viruses, which can only be achieved if the molecular biology and the epidemiology of the viruses are fully understood. Their geographical distribution largely determines the incidence and prevalence of neurological diseases caused by these viruses. In America, for example, the annual incidence of viral meningitis is higher than the incidence of meningitis caused by all other pathogens, reaching more than 10,000 cases annually up to 75,000 cases [1-3].

Viral infections of the central nervous system have been emerging. Re-emerging infections and the clinical spectrum mainly depend on certain factors, which include the viral genomic constitution, the geographical location of the viral vector, host immune status, and some environmental factors. However, they are commonly misdiagnosed and their etiology frequently missed, partly due to a lack of proper diagnostic apparatus and partly due to poor understanding of the viral biology and their epidemiology [2]. The etiologic agents of viral CNS infections also vary according to geographical locations. Herpes simplex viruses (HSVs) are the most common pathogens isolated among both children and adults in the United States (US), Australia, and Italy, while in Southern Vietnam, the Japanese encephalitis virus (JEV) is the most frequent cause of viral encephalitis among children. In some parts of India, enteroviruses (EVs) were identified as the leading cause of viral encephalitis, while HSVs remained the most prevalent pathogen in eastern India among both children and adults [3]. Therefore, understanding the epidemiology of neurotropic viruses is paramount in designing the targeted intervention.

In this review, we focus on the molecular biology and the epidemiology of the different viruses that can potentially cause neurological diseases. We review the current understanding of the different neurotropic viruses from the different viral families and their neurotropic members to the structure and composition of the viral genome, the geographical distribution of the different viruses, and then down to the worldwide prevalence and incidence. We also aim to outline the morbidity and mortality profile of the different neurotropic viruses and the contributing epidemiological factors in the spread and outbreaks of these viruses. Therefore, the overall objective of the paper is to give a complex picture of the different neurotropic viruses, elucidating on their genetic composition, viral replication, geographical location, and their demographic profile, which will immensely help in understanding the mechanism employed by the viruses during neuroinvasion and neurovirulence.

## Review

Picornaviruses

Molecular Biology

Picornaviruses, one of the largest virus families, are small (30 nm in size), single-stranded, non-enveloped ribonucleic viruses that contain 29 genera with EVs and Parechoviruses (PeVs) as human pathogenic genera. The genus EV is divided into 13 species designated as EV A-J and Rhinoviruses (RVs) A-C, of which seven (EV A-D and RV A-C) are human pathogens, and the remaining six (EV E-J) were shown to infect only animals [1, 4-6]. The subgroups of EVs include the Polioviruses, Coxsackieviruses A and B, Echoviruses, numbered EVs, and RVs, whereas the genus PeV is divided into two species, Parechovirus A and B [1, 6]. The Parechovirus A species consists of 16 genotypes, and they are designated as human Parechoviruses (HPeVs) 1 - 16. In contrast, Parechoviruses B species consists of four genotypes, 1 - 4, which are known to infect rodents and are previously known as the Ljungan virus [4, 7].

The viral genome of Picornaviruses is a single, open reading frame with highly structured untranslated regions (UTRs) at the 5- and 3-ends, it is uncapped, and the 5′-end is covalently coupled to the viral protein 3B, hence called viral protein genome (VPg)-linked. The setting of the open reading frame is similar in all Picornaviruses, with some differences between genera and some species. The genus EV consists of a simple viral capsid constructed in 60 repeating protomers, each of which contains four structural proteins (VP 1 - 4), that collectively form an icosahedral shell [1, 5-6].

Epidemiology

The human pathogenic Picornaviruses (EVs and PeVs) constitute the major causes of aseptic meningitis in children, especially in neonates and young infants [4]. Human EVs, which are more prevalent than HPeVs, has a worldwide distribution with the highest incidence in the summer and autumn and mostly transmitted feco-orally among children [1, 4]. In the United Kingdom (UK) and Ireland, EVs constitute about 50% of the reported cases, and 92% of EV meningitis occurred in infants less than three months, with an annual incidence of EV meningitis of 0.79 per 1,000 live-births [8]. The estimated incidence of EV meningitis in the US in infants less than three months varies from 3.2% to 50%; about 40% of the children below the age of 12 months and 90% of the children below the age of two years had EV infections in Norway [4]. Thong et al. found out that the peak incidence of EV infection in Singaporean children was seen in infants less than one year [9]. The most predominant genotypes causing meningoencephalitis in children were shown to be Echoviruses, Coxsackieviruses, and numbered EVs [5, 10-12]. HPeVs form an important cause of viral meningitis in infants caused majorly by the most pathogenic type, HPeV-3. In the UK and Ireland, the annual incidence of HPeVs meningitis was 0.04 per 1,000 live-births [6, 8].

Arboviruses

Molecular Biology

As the name implies, arboviruses are arthropod-borne viruses with a worldwide distribution that encompasses hundreds of heterogeneous groups of about 537 viruses. They are also called tick-borne viruses. Most arboviruses have a ribonucleic acid (RNA) genome that has higher genetic plasticity and mutational rate [2, 13-14]. Clinically, arboviruses are classified into four groups:

1) Togaviridae: The clinically important viruses in this group belong to the genus alphavirus which consists of the following species: Eastern equine encephalitis virus (EEEV), Western equine encephalitis virus (WEEV), and Venezuelan equine encephalitis virus (VEEV), as well as the Mayaro virus (MAYV), Una virus (UNAV), and Chikungunya virus (CHIKV). The first groups are called New World alphaviruses, while the second groups are called Old World. Alphaviruses are small, enveloped viruses with a single-stranded, positive-sense RNA genome that has 5’ cap and 3’ poly-A tail with two open reading frames that are translated into two polyproteins comprising structural and nonstructural proteins, respectively [13-14].

2) Flaviviridae: The word flavus comes from the Latin word meaning yellow, as observed in the yellow fever virus infection. Flaviviruses are very diverse, consisting of the world's clinically most important viruses and consist of the following species: Japanese encephalitis virus (JEV), tick-borne encephalitis virus (TBEV), and Powassan encephalitis virus (POWV), as well as other mosquito-borne viruses, such as the Dengue virus (DENV), yellow fever virus (YFV), West Nile virus (WNV), St. Louis encephalitis virus (SLEV), and Zika virus (ZIKV). Hence, broadly, there are two forms of flaviviruses: tick-borne and mosquito-borne flaviviruses. They are enveloped structured, single-stranded, positive-sense RNA viruses with a single open reading frame which is translated into a polyprotein that is cleaved into mature structural and nonstructural polypeptides by viral and host proteases [14].

3) Bunyaviralis: The family Bunyaviralis consists of three main genera - Nairovirus with a species of Crimean-Congo hemorrhagic fever (CCHF); orthobunyaviruses with species of California encephalitis virus (CEV), Jamestown Canyon virus (JCV), and La Crosse encephalitis (LACV); and phleboviruses with species of Toscana virus (TOSV) and Rift Valley fever virus (RVFV). The Bunyaviruses are enveloped particles of about 80 - 120 nm containing a negative-sense, single-stranded RNA genome. They are tripartite, containing three segments, each in its nucleocapsid structure [1, 13].

4) Reoviridae: The family Reoviridae is divided into two main subfamilies with many genera: Sedoreovirinae (with the genera of Orbivirus, Cardoreovirus, Rotaviruses, and Phytoreovirus) and Spinareovirinae (with genera of Orthoreovirus, Aquareovirus, and Dinovernavirus). The genus Orbivirus is the most clinically important genus with species of Colorado tick fever virus (CTFV). They are double-stranded segmented RNA viruses. The virion is nonenveloped and divided into 12 segments, with each viral family utilizes distinct strategies for infection and replication [1, 14].

Epidemiology

Many arboviruses have been identified worldwide, with the global distribution determined mainly by the biology of the vectors and the animal reservoirs. Transmission occurs throughout the year and is affected by the changes in environmental conditions, such as temperature, rainfall, humidity, and vegetation [1, 14]. Arboviruses or tick-borne viruses are viruses that can infect humans via arthropod vectors, including mosquitoes, ticks, and sand flies (Phlebotomus sp.). The route of transmission can be horizontal, vertical, including mother to child transmission, and nosocomial through either blood transfusion or organ transplant [13].

1) Togaviridae/Alphaviruses: The transmission of alphaviruses causing encephalitis typically occurs between mosquitoes and birds (EEEV and WEEV), mosquitoes and rodents (VEEV enzootic cycle), or mosquitoes and horses (VEEV epizootic cycle). When humans contract the infection, it rapidly progresses to encephalitis with a fatality of about 1% in cases of VEEV and WEEV and 50% to 75% in cases of EEEV. Alphaviruses have a global distribution and are typically classified into Old and New Worlds. The New World alphaviruses are found in the American continent and are the ones causing encephalitis, whereas the Old World are found in Europe, Asia, and part of Africa with few members, like CHIKV, causing encephalitis. The most important neurotrophic alphavirus is VEEV, which caused many outbreaks in South, Central, and North America [15]. CHIKV is now becoming a re-emerging epidemic with cases reported in more than 45 countries and has caused severe diseases with neurological complications affecting all age groups [15-16]. CHIKV is transmitted to humans through mosquito vectors where the vertebrate animals serve as the reservoirs in epizootic cycles, but in current epidemics, humans serve as the reservoirs. In La Reunion Island and the Caribbean CHIKV outbreaks, the incidence of encephalitis was reportedly 187 per 100,000 infants [14].

2) Flaviviridae: Flaviviruses are transmitted by both ticks and mosquitoes, hence classified into tick-borne flaviviruses and mosquito-borne flaviviruses. The former includes the tick-borne encephalitis virus (TBEV) and Powassan encephalitis virus (POWV), while the latter includes JEV, DENV, WNV, YFV, SLE, and ZIKV. Tick-borne flaviviruses have been considered medically important as they cause about 10,000 to 15,000 human cases every year in Europe and Asia [15]. Mosquito-borne flaviviruses are considered as the most important neurotropic flaviviruses [16].

Tick-borne encephalitis (TBE), caused by TBEV, is mainly a central nervous system infection that is transmitted to humans by the bites of ticks. The incidence is progressively increasing in European and Asian countries with serious neurological complications. POWV is a tick-borne flavivirus, similar to TBEV, caused by the bite of Ixodesscapularis, Ixodes cookei (I. cookei), and Ixodes marxi ticks (I. marxi ticks) and transmission occurs very quickly (approximately 15 minutes after tick attachment) [17].

Japanese encephalitis (JE) virus is associated with high morbidity and mortality in infants, and about 2 billion people were reported to be affected annually in tropical and subtropical countries. Most of the cases are reported annually from the People’s Republic of China (PRC), Korea, Japan, Indonesia, Cambodia, Thailand, Vietnam, Malaysia, and countries belonging to the Indian subcontinent, and parts of Oceania with at least 700 million potentially susceptible children [18]. Ornithophilic mosquitoes generally transmit it; however, it can also be transmitted via blood transfusion or transplacentally to the fetus during pregnancy [14-15].

West Nile virus (WNV), which was identified first in the West Nile subregion of Uganda, is now endemic in temperate and tropical regions throughout the world, causing yearly outbreaks of encephalitis, with a mortality rate of 5% to 10%. Ornithophilic mosquitoes generally transmit JEV; however, it can also be transmitted via blood transfusion or transplacentally to the fetus during pregnancy [14]. WNV has caused sporadic outbreaks in Africa, the Middle East, Asia, and Australia. Most WNV infections are subclinical, and less than 1% of the affected will develop neurological complications ranging from meningitis, encephalitis, or meningoencephalitis [15].

Dengue virus (DENV) has a worldwide distribution resulting from human migration, uncontrolled urbanization, armed conflict, inadequate waste and water management, and unsustainable vector control. It is endemic in more than 100 countries and is transmitted to humans by the female mosquitoes of the genus Aedes (Ae), mainly Ae. aegypti and Ae. albopictus, during a blood meal. It is divided generally into four genetically related but antigenically different groups, designated as DENV 1 - 4 groups, which are widely circulating in both urban and peri-urban environments [13-14].

Yellow fever virus (YFV) is the cause of the clinically important disease of public health concern called yellow fever infection. It is found in all continents but of more public health importance in the African and American subcontinents. It causes large epidemics in Africa and America with high morbidity and mortality. The incidence in West Africa is about 50 cases per 100,000 population, indicating the high infectivity of the virus in the region compared to the incidence of five cases per 100,000 population in South America. Paradoxically, the mortality rate in South America is higher than that in Africa, likely due to some genetic variability [13].

St. Louis encephalitis virus (SLEV), a mosquito-borne flavivirus, oc­curs in North, Central, and South America and is one of the most important arbovirus infections in North America. It accounts for approximately 35% - 60% of aseptic meningitis in all symptomatic cases in children [13, 15].

Zika virus (ZIKV) is a mosquito-transmitted flavivirus, that was discovered in Uganda in 1947, which later caused outbreaks in Micronesia, French Polynesia, and South and Central America. It was first isolated in humans in 1952, with only sporadic cases reported in Africa and parts of Asia. Subsequently, from 2001 onward, many outbreaks of ZIKV infections were reported in many parts of the world. In 2016, the World Health Organization announced that ZIKV was an international emergency [13]. To date, cases of ZIKV has been reported in 48 American countries and dependent territories, including imported cases in the United States, Canada, and Europe [13-14].

3) Bunyavirales: Bunyaviruses have a wide range of zoonotic life cycles. Mosquitoes transmit some members, like CEV and LACV, and therefore, cycle between mosquitoes and small mammals [14]. They are usually endemic in both the western and eastern parts of the US. They cause significant morbidity and mortality as about 75 cases of meningitis, encephalitis, and meningoencephalitis every year are attributed to CEV and LACV, with the majority of the cases caused by LACV. In Kenya, Somalia, Tanzania, Saudi Arabia, and Yemen, RVFV was reported to have caused severe outbreaks, with about 20,000 cases and more than 500 deaths. RVFV is also transmitted by mosquitoes or via extensive contact with blood, milk, and body tissues from infected livestock. Other members of Bunyaviruses, like TOSV, are transmitted by sandflies, and in Europe and North Africa, about 100 to 200 cases of meningoencephalitis each summer have been attributed to these members. Canines have been identified as a potential natural reservoir for TOSV [14].

4) Reoviridae: The clinically important member of this family is the Colorado tick fever virus (CTFV), which cycles among many species of rodents and Rocky Mountain wood ticks. Between 2002 to 2012, about 83 cases of CTFV infections were reported mainly from the western parts of the US and Canada [14].

Paramyxoviruses

Molecular Biology

These are broad family members of Paramyxoviridae that infect humans and animals. The three clinically important genera that are known to cause neurological diseases are 1) Rubulavirus with the mumps virus as a neurotropic virus, 2) Morbillivirus with measles virus as clinically important species, and 3) Henipavirus with Nipah virus (NiV) and Hendra virus (HeV) as neurotropic members [19-20].

The mumps virus is an enveloped, non-segmented, single-stranded RNA virus with pleomorphic structures containing an inner ribonucleoprotein core structure with two protein molecules, one of which serves as RNA-dependent RNA polymerase. Two surface glycoproteins are responsible for the neuraminidase, hemagglutination, and fusion properties of the mumps virus that facilitate adsorption of the virion to host cells and penetration of the genetic material into the cells [1, 19, 21].

The measles virus is a single-stranded, negative-sense RNA virus enclosed within a lipid capsule. It has a non-segmented genome of about 16,000 nucleotides linearly with six genes that encode eight viral proteins (six structural and two nonstructural proteins). The six structural proteins are haemagglutinin protein, fusion protein, nucleocapsid protein, phosphoprotein, matrix protein, and large protein. It is spherical with a diameter of about 100 - 200 nm and shows pleomorphism [3].

Henipaviruses, including both NiV and HeV, are enveloped, single-stranded, nonsegmental, and negative-sense RNA viruses that represent emerging zoonotic infections that present with acute encephalitis and respiratory distress syndrome [22-23].

Epidemiology

The mumps virus (MuV) is a species of the genus Orthorubulavirus of the family Para­myxovirus affecting the CNS and glands. It is isolated in the saliva, cerebrospinal fluid, blood, breast milk, infected tissues, and urine. Transmission is through con­tact with respiratory secretions, saliva, direct contact, or through fomites, like bedding or doorknobs. It causes high cases of encephalitis in unvaccinated infants [20, 24]. Cases of vaccine-associated mumps meningitis have been reported [1]. The incidence ranges from one to 10 cases per 10,000 doses, with meningitis occurring 15 to 35 days after vaccination. However, according to Campbell et al., there is no proof that the encephalitis observed in vaccinated children was due to the mumps component of the vaccine [25]. The fourfold rise in mumps S titer, as described by Crowley et al. in a 14-month-old girl, could reflect successful immunization [26].

Measles virus (MV), a member of the genus Morbillivirus, remained a leading cause of morbidity and mortality in the developing world, with an estimated 122,000 deaths in 2012. MV infection can cause severe debilitating CNS complications. It has been suggested up to 30% of cases with approximately 1:6,000 cases are complicated by more severe MV encephalitis. Transmission is via airborne droplets or small-particle aerosols that remain suspended in the air, direct contact with infected secretions, and less commonly, contact with contaminated fomites [3].

Henipaviridae consists of two important viruses, the Nipah virus (NiV) and Hendra virus (HeV). The natural reservoir of both viruses is Pteropid bats, which harbor the viruses but do not show clinical illness. Virus transmission from bats to domestic animals is thought to be through pasture or feed contaminated by bat urine, feces, or other excretions. Transmission of HeV to humans has been invariably associated with close contact with ill horses while transmission of NiV, as reported from Bangladesh, is mainly through date palm sap contaminated with bat secretions. Human-to-human transmission of NiV also occurs [27-28]. 

Arenaviruses (lymphocytic choriomeningitis viruses)

Molecular Biology

Lymphocytic choriomeningitis virus (LCMV) belongs to the family Arenaviridae, and it is the only member of the family that causes diseases in humans. It is an enveloped, single-stranded RNA virus with the genome containing two segments, the small (S) and the large (L) segments. The virus produces one nucleocapsid protein and two glycoproteins. The S segment encodes the nucleocapsid protein (NP) and the glycoprotein precursor (GPC). The L segment encodes the viral RNA-dependent RNA polymerase (L) and the small zinc finger-like protein (Z). More than 30 strains have been isolated with some enzymatic activities of polymerase and transcriptase functions [3, 22].

Epidemiology

LCMV is a member of the Arenaviridae family, with its primary host being the house mouse (Mus musculus). However, it has also been detected in other rodent species, such as hamsters, rats, and guinea pigs. The greatest risk of transmission occurs in laboratory workers, pet owners, and individuals living under impoverished and non-hygienic circumstances [1]. It is found in Europe, Asia, American continents, and Africa. Serological studies indicate that approximately 5% of the human population in the United States have LCMV antibodies, 3.3% in Inland city of Argentina, 4% among inhabitants of Nova Scotia, 1.7% in the Community of Madrid in Spain, 3.5% in the inner city of Birmingham, and 36% in Croatia Island of Vir [27, 29].

Herpes family viruses

Molecular Biology

Meningitis and encephalitis have been associated with HSV infection and commonly results in severe consequences with severe developmental impairment and disability [30]. The members of herpes family viruses that are shown to be neurotropic include HSV types 1 and 2, varicella-zoster, Epstein-Barr virus, and cytomegalovirus [1]. Both HSV-1 and 2 belong to the family of Herpesviridae and have the propensity of establishing latency after primary infection with subsequent reactivation [31]. All herpesvirus genomes were shown to contain microsatellites, which are multiple sites with short tandem repeats of one to six nucleotide motifs and homopolymers as abundant microsatellites class [32]. There are 84 recognized unique protein-coding open reading frames (ORFs) and several RNA transcripts that are not proven to encode proteins [33].

Herpes simplex virus type 1 (HSV-1): HSV-1 is an enveloped, large deoxyribonucleic acid (DNA) virus that typically infects at epithelial surfaces with the subsequent establishment of latent infection in the sensory neurons. The virus has a double-stranded DNA genome of about 152 kb with two unique regions (long of about 108 kb and short of about 13 kb), and large inverted repeats flank each with a large number of simple sequence repeats (SSRs), also known as variable number tandem repeats (VNTRs) or reiterations. About 84% of the HSV-1 genome consists of protein-coding regions, which contain about 60% of the total SSR loci [34-35].

Herpes simplex virus type 2 (HSV-2): The HSV-2 is a linear double-stranded DNA virus of about 154 kb. The genome also contains two unique regions as in HSV-1, long and short, but is bounded by a pair of terminal repeat and internal inverted repeat elements and over 74 open reading frames [32].

Varicella-zoster virus (VZV): Varicella-zoster virus (VZV), also known as human herpesvirus 3, is a double-stranded DNA virus that naturally infects humans, with no animal reservoir. Primary infection usually occurs during childhood which causes varicella (chickenpox), it then becomes latent in the spinal and cranial ganglia. When the cellular immunity to the virus goes down with advancing age or in immunocompromised individuals, it then becomes reactivated and causes zoster (shingles) [36-37]. The genome contains about 125,000 base pairs, including 70 open reading frames (ORFs). It has single nucleotide polymorphisms (SNPs) which, upon analysis, reveals various clades of VZV strains [38].

Epstein-Barr virus (EBV): Epstein and Barr are names of physicians who discovered virus particles in cultured lymphoblasts from Burkitt’s lymphoma (BL) in 1964 [39]. It is one of the human herpesviruses, sometimes referred to as human herpesvirus type 4 (HHV-4). The EBV is an enveloped, double-stranded, and linear DNA virus with its virions surrounded by a protein capsid. Between the capsid and the envelope lies a protein tegument that contains glycoproteins, which serves for cell tropism, host range, and receptor recognition. The virions measured about 120 - 180 nm in diameter and contain about 100 genes. Based on the nuclear antigen, EBV is divided into two subtypes distinguished by their restriction endonuclease digestion patterns and the capability of spontaneous lytic transformation [39].

Cytomegalovirus (CMV): Human CMV, a member of the herpesvirus group, is a double-stranded, linear DNA virus. The nucleic acid and the protein helically form a core covered by a protein capsid containing about 162 capsomeres, the majority of which are hexagonal in structure with a few pentameric. The diameter of the capsid is about 100 nm which is surrounded by a tegument, and when enclosed by a lipid bilayer envelope (which contains peplomers), the diameter reaches 180 nm. The length of the virion is about 230 - 240 kb [40-41].

Epidemiology

Children and immunocompromised individuals are the most vulnerable of herpes simplex meningoencephalitis. In the US, about 1,500 cases of HSV infection occur annually. The mortality rate of neonatal HSV infection, which is commonly contracted during delivery, can be as high as 80%, if left untreated, with serious neurologic complications [42-43]. In the Western world, HSV is the commonly isolated pathogen in both sporadic and outbreaks of encephalitis, with an incidence of one to three cases per million populations annually. The occurrence of HSV encephalitis in post-surgical procedures was reported, indicating a nosocomial infection [27]. Worldwide, HSV is the most common cause of sporadic encephalitis, and the distribution is not determined by seasonal variation, immune status of a community, or environmental conditions. The mode of transmission is through contact with an infected epithelial surface of genitalia, gastrointestinal tract, or eyes and transmitted to the CNS via sensory nerve ganglia [35, 44]. In a study by Ward et al., most of the cases of HSV CNS infection with neurological sequelae were found in infants less than two years with an estimated incidence of HSV CNS infection in the UK as one in 64,000/year in children aged two to 11 months and one in 230,000/year in children aged 12 - 35 months [45]. About 33% of neonatal HSV infections occur as CNS infections, and in the US, most of the HSV infections beyond the first three months of life occur as herpes simplex encephalitis [31]. HSV-1 is more prevalent than HSV-2, and both can cause latent infection and reactivation [32, 35].

Varicella-zoster virus (VZV) has a worldwide distribution but more endemic in temperate countries. It is highly contagious in an overcrowded area, and transmission is via airborne droplet and contact with infected skin, the most common mode of transmission in children. The incidence is highest in preschool children aged one to four years with the distribution determined by seasonal variation, commoner during the winter and spring, and environmental conditions. Congenital VZV infections were reported in about 2% of infected pregnant women [36-37].

Epstein-Barr virus (EBV) was identified in 1964 using an electron microscope from the cultures of African Burkitt lymphoma cells [39]. It is distributed globally with no clear seasonal variation. The mode of transmission in children is mainly due to close contact with secretions from an infected person or contact with infected materials, such as toys, utensils, neuroinvasive, or feeding materials. Other modes of transmission include blood transfusion, transplacentally from mother to child, and after organ transplant. The risk factors for transmission include low socioeconomic status, overcrowding, and young age [46].

CMV is highly endemic in most of the world, and the distribution has no seasonal variation. The seroprevalence was estimated to be between 30% - 100% in a different part of the world. The seroprevalence in China is about 0.6% - 8.5% in the newborn and 58% - 84% in infants [47-48]. The mode of transmission varies from direct contact to nosocomial and transplacental transmission from mother to fetus, resulting in congenital cytomegalovirus with an estimated incidence in developed countries that ranges from 0.6% to 0.7% of all live births. The risk factors for transmission include low socioeconomic status, sexual activity, overcrowding, and increasing birth order [47-49].

## Conclusions

This review provided a current understanding of the molecular, epidemiologic, and biologic characteristics of some neurotropic viruses. The morbidity and mortality data of many viruses are provided herein. Various factors responsible for the spread and outbreaks of some viruses are also outlined. Moreover, the mode of viral transmission from the animal reservoir to humans has been explained. This should help the scientific community to better understand the different viral genomes and, therefore, to develop therapeutics and interventional measures. The study has some limitations, as only published data were used and was restricted only to molecular biology and epidemiology with no discussion on the neuropathogenesis. Further studies are required for exploring the relationship between disease causation and viral structure and the role played by the ecological, demographic, and environmental factors in the process.
